# Broad laboratory testing does not show utility for telogen effluvium: A retrospective analysis of 22-million laboratory results using TriNetX

**DOI:** 10.1016/j.jdin.2025.12.013

**Published:** 2026-01-10

**Authors:** Zachary J.K. Neubauer, Shari R. Lipner

**Affiliations:** a Sidney Kimmel Medical College, Thomas Jefferson University, Philadelphia, Pennsylvania; b Department of Dermatology, Weill Cornell Medicine, New York, New York

**Keywords:** hair loss, laboratory testing, retrospective, telogen effluvium, TriNetX

*To the Editor:* Telogen effluvium (TE) is a self-limited hair loss condition following an inciting event, such as systemic disease, medications, or psychological stress.^1^ A broad array of laboratory tests are often ordered to workup TE, which may be fruitless and unnecessarily costly.^1^^,^^2^ We aimed to evaluate laboratory testing utility for identifying TE etiologies using a large national database.

Using TriNetX’s database, laboratory results for 26 blood tests were compared between females with versus without TE after propensity-score matching. Odds ratios and 95% confidence intervals were calculated by comparing the number of abnormal laboratory results between cohorts. Separately, mean laboratory values were compared with a *t* test, with *P* values and standard mean differences (SMD) reported. Bonferroni correction was used to account for multiple hypothesis testing (Supplementary Table I, Supplementary Methods, available via Mendeley at https://data.mendeley.com/datasets/485bhvbyvf/3).

We included 2,059,756 patients with 22.8-million test results. There were 13,732 patients with TE propensity-score matched to an equivalent number of controls. After propensity-score matching, all demographic comparisons between TE and control cohorts had *P* > .96 (Table I). Comparing abnormal result counts found that patients with TE more often had abnormal aspartate aminotransferase (AST) (12.1% vs 9.0%, odds ratio = 1.39; 95% confidence interval, 1.18-1.63) results. Comparing mean laboratory values found that TE versus control subjects had lower dehydroepiandrosterone sulfate (SMD, −0.550), total (SMD, −0.516) and free (SMD, −0.409) testosterone levels, and higher creatinine (SMD, 0.066), calcium (SMD, 0.093), AST (SMD, 0.121), iron (SMD, 0.174), and iron saturation (SMD, 0.208) levels (all *P* < .01) (Table II).

**Table I:** Demographics of patients with TE versus healthy control patients before and after propensity score matching

Demographic	Before match				After match			
	TE	Controls	SMD	*P* value	TE	Controls	SMD	*P* value
Totals	13,735	2,046,021	-	-	13,732	13,732	-	-
Age at index, y	45.2 ± 19.8	47.6 ± 18.9	0.126	<.01	45.2 ± 19.8	45.2 ± 19.8	<0.01	.972
Hispanic or Latino	1280 (9%)	146,155 (7%)	0.079	<.01	1280 (9.3%)	1280 (9.3%)	<0.01	1.000
Asian	912 (7%)	134,507 (7%)	<0.01	.7561	909 (6.6%)	907 (6.6%)	<0.01	.961
Black or African American	958 (7%)	284,722 (14%)	0.228	<.01	958 (7.0%)	960 (7.0%)	<0.01	.962
White	10,019 (73%)	1,427,723 (70%)	0.070	<.01	10,019 (73.0%)	10,018 (73.0%)	<0.01	.989
Asymptomatic menopausal state	829 (6%)	97,930 (5%)	0.055	<.01	829 (6.0%)	830 (6.0%)	<0.01	.980

*SMD*, Standard mean difference; *TE*, telogen effluvium.

**Table II:** Post propensity-score matched comparison of mean values and risk of abnormal result for laboratory testing performed within 90-days after diagnosis of telogen effluvium versus healthy controls

Laboratory test	Number of abnormal results comparison	Comparison of mean laboratory values			
	Odds ratio (95% CI)∗	TE values	Control values	*P* value†	SMD
Alanine aminotransferase (U/L)	1.10 (0.93-1.30)	21.6 ± 19	20.9 ± 20.6	.884	0.034
Alkaline phosphatase (U/L)	1.24 (1.07-1.45)	76.6 ± 37.8	75.5 ± 37.1	1.000	0.030
Antinuclear antibody‡ (titer 1:N)	0.59 (0.34-1.02)	316.9 ± 356.8	§	-	-
Aspartate aminotransferase (U/L)	1.39 (1.18-1.63)	23.1 ± 14.4	21.5 ± 11.9	<.01	0.121
Calcium (mg/dL)	1.02 (0.65-1.60)	9.5 ± 0.5	9.4 ± 0.5	<.01	0.093
Chloride (mmol/L)	1.26 (0.86-1.85)	103.5 ± 6.7	103.5 ± 5.1	1.000	−0.008
Creatinine (mg/dL)	0.83 (0.66-1.06)	0.8 ± 0.7	0.8 ± 0.4	<.01	0.066
Dehydroepiandrosterone sulfate (DHEA-S) (μg/dL)	װ	141.5 ± 101.4	199.2 ± 108.1	<.01	−0.550
Direct bilirubin (mg/dL)	1.53 (0.81-2.86)	0.2 ± 0.1	0.1 ± 0.1	.676	0.134
Erythrocytes (106/μL)	0.91 (0.71-1.16)	4.4 ± 0.6	4.4 ± 0.7	1.000	0.021
Ferritin (ng/mL)	0.40 (0.29-0.55)	73.2 ± 98.3	73.2 ± 190.1	1.000	0.000
Free thyroxine (ng/dL)	0.95 (0.51-1.77)	1.2 ± 0.5	1.1 ± 0.4	.520	0.071
Hemoglobin (g/dL)	1.04 (0.86-1.26)	13.2 ± 1.3	13.2 ± 1.3	1.000	−0.025
Indirect bilirubin (mg/dL)	1.50 (0.82-2.76)	0.5 ± 0.3	0.4 ± 0.3	1.000	0.070
Iron (μg/dL)	0.26 (0.17-0.38)	86.1 ± 37.6	79 ± 43.9	<.01	0.174
Iron binding capacity (μg/dL)	0.56 (0.35-0.89)	977.2 ± 14,170.4	1970.7 ± 18,395.3	1.000	−0.061
Iron saturation (%)	0.41 (0.29-0.58)	25.2 ± 11.7	22.6 ± 13.1	<.01	0.208
Leukocytes (103/μL)	1.46 (1.06-2.03)	15.7 ± 202.2	13 ± 162.4	1.000	0.015
Platelets (103/μL)	0.99 (0.70-1.39)	270.4 ± 71	272.2 ± 70.5	1.000	−0.026
Potassium (mmol/L)	1.04 (0.87-1.26)	4.2 ± 0.5	4.2 ± 0.4	.520	−0.034
Sodium (mmol/L)	1.11 (0.85-1.45)	139.4 ± 2.5	139.4 ± 2.4	1.000	−0.017
Testosterone (ng/dL)	0.92 (0.50-1.68)	29.4 ± 73.9	106.4 ± 198	<.01	−0.516
Testosterone free (pg/mL)	0.17 (0.07-0.42)	3.3 ± 8.7	9.3 ± 18.9	<.01	−0.409
Thyrotropin (m(IU)/L)	1.00 (0.81-1.23)	3 ± 28.3	3 ± 26.8	1.000	−0.000
Total bilirubin (mg/dL)	0.82 (0.67-1.01)	0.5 ± 0.3	0.5 ± 0.3	.702	0.038
Zinc (μg/mL)	װ	19.7 ± 64.5	5 ± 16.4	1.000	0.312

*CI*, Confidence interval; *SMD*, standard mean difference; *TE*, telogen effluvium.

∗ Bonferroni adjusted CIs were calculated by using an adjusted significance threshold (α = 0.05/26) for determining the interval bounds.

† *P* values resulting from the *t* test of unequal variance were multiplied by the number of tests performed (26) to result in the Bonferroni adjusted *P* value. Modified *P* values exceeding 1.000 were expressed as 1.000.

‡ Abnormal results for “Antinuclear Antibody” include positive test results or titer results above 1:80.

§ Because ≤10 control patients had antinuclear antibody titer results, TriNetX obfuscated the number of patients, preventing *t* test comparison of mean laboratory results.

װ Because ≤10 patients had abnormal values, TriNetX obfuscated the number of patients, preventing the calculation of an odds ratio.

In this large cohort study, laboratory testing could not effectively identify TE etiology, as only AST showed significant mean value differences coinciding with increased abnormal result odds. However, with minimal SMDs and no known association between AST changes and TE,^1^ these results are likely not clinically meaningful. Similarly, a retrospective study of 1342 patients with TE analyzed 90-days after diagnosis found that 72.7% of laboratory abnormalities were mild, had low clinical significance, and were unlikely to impact management. A financial analysis of the 16,381 laboratory results from those same patients estimated a cost of $2435.05 to find one highly/severely abnormal laboratory result.^3^ A retrospective study of 138 patients with nonscarring hair loss, including 18 with TE, analyzed for ferritin, zinc, vitamin D, and thyroid hormone, reported that over half (*n* = 70) had laboratory abnormalities. However, there was no difference in hair density change following supplementation regardless of whether laboratory values did (*P* = .48) or did not (*P* = .67) normalize,^4^ suggesting that supplementation does not improve TE-associated hair loss.

Limitations include retrospective design, potential miscoding, and inability to assess laboratory documentation or TE duration. Laboratory summary statistics may not capture clinical variations. Associations cannot be attributed to causal relationships. Patient counts for antinuclear antibodies, dehydroepiandrosterone sulfate, and zinc testing fell below TriNetX’s minimum report threshold, preventing analysis. Bonferroni correction decreases type I error but increases type II error.

In sum, in this study analyzing >22-million laboratory results, no test had utility in identifying TE etiology. Although specific laboratory testing may be indicated for suspected TE and concurrent symptoms (ie, thyroid function and weight change), we discourage laboratory testing patients with a negative review of systems. If patients request testing, dermatologists may reassure patients of TE’s self-limiting prognosis and schedule clinical follow-up. Large prospective studies are needed to replicate these findings.

## Conflicts of interest

Dr Lipner has served as a consultant Moberg Pharmaceuticals and BelleTorus Corporation. Author Neubauer has no conflicts of interest to declare.
